# Modeling Graphene–Polymer Heterostructure MEMS Membranes with the Föppl–von Kármán Equations

**DOI:** 10.1021/acsami.2c21096

**Published:** 2023-02-07

**Authors:** Katherine Smith, Aidan Retallick, Daniel Melendrez, Aravind Vijayaraghavan, Matthias Heil

**Affiliations:** † Department of Materials and National Graphene Institute, The University of Manchester, ManchesterM13 9PL, U.K.; ‡ Department of Mathematics, The University of Manchester, ManchesterM13 9PL, U.K.

**Keywords:** MEMS, membrane, Föppl−von Kármán, pressure sensor, graphene

## Abstract

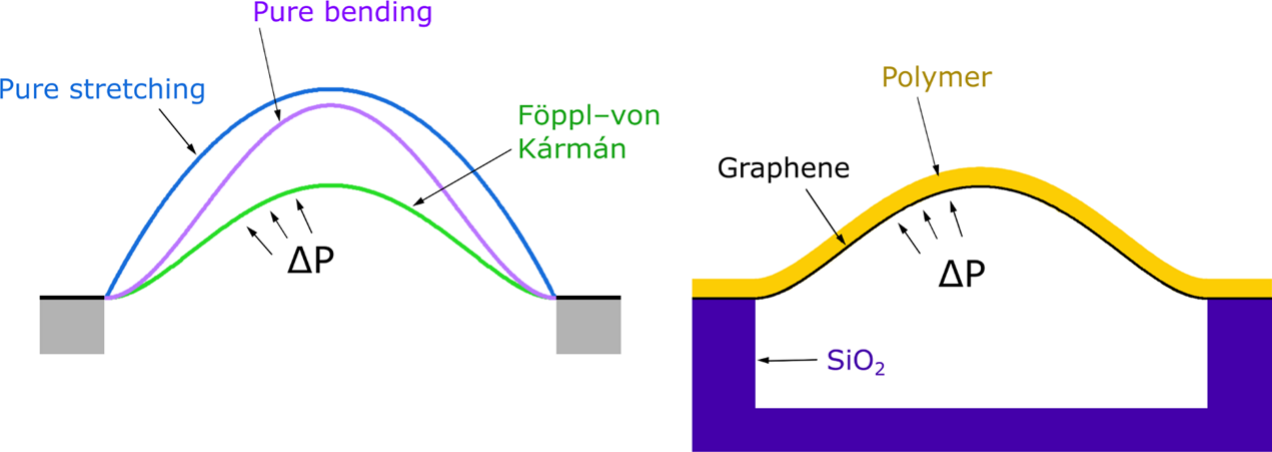

Ultra-thin graphene-based membranes have shown significant promise for high-performance nano-electro-mechanical (NEMS) devices. The key challenge in the modeling of such membranes is that they often operate in deflection regimes where the assumptions or approximations of “pure bending” or “pure stretching” are not satisfied. We present a model of graphene–polymer heterostructure (GPH) NEMS membranes based on Föppl–von Kármán (FvK) equations which take into account both bending and stretching forces. The experimental GPH membrane shape obtained through atomic force microscopy topography mapping is compared to the inflation shapes predicted by FvK-based finite element method simulation, and they show excellent agreement with each other. When the GPH membranes are deflected under pressure in a capacitive pressure sensor configuration, the effectiveness of this model is further exemplified through accurately predicting the capacitance change of deflecting GPH membrane devices at varying pressures. This model serves as a powerful new tool in the design and development of graphene-based NEMS devices, being able to predict the performance of graphene NEMS devices or to aid in the design of device geometries to match required performances.

## Introduction

The extraordinary properties of graphene, the two-dimensional (2D) carbon allotrope, such as its high Young’s modulus^1^ (1 TPa), negligible bending rigidity,^2^ and high electrical conductivity make it an ideal candidate as a micro- and nano-electromechanical system (MEMS/NEMS) actuator, for example, in capacitive pressure sensors and microphones. The most favorable manufacturing process of graphene for such devices is chemical vapor deposition (CVD) on copper foil, as it produces high-quality single-layer graphene in large, uniform monolayer sheets.^3^ Defect-free polycrystalline CVD graphene is impermeable to all gases,^4^ which makes it viable as a barrier in high-pressure applications; however, the growth of entirely defect-free CVD graphene remains a challenge.^5^ Furthermore, the atomic thinness of graphene (∼0.335 nm) renders it highly susceptible to rips, wrinkles,^6^ and stiction issues, as well as damage and contamination^7^ during transfer and manufacturing processes, drastically reducing device yield and performance.^8,9^ In recent years, it has been demonstrated that these challenges can be overcome with the deposition of a uniform, conformal nanometer thickness support polymer such as 2-chloro-*p*-xylylene (parylene-C) to form a graphene–polymer heterostructure (GPH) NEMS membrane.^10^

An ultra-thin support layer of parylene-C has been shown to be sufficient to achieve 100% yield of intact and functioning membranes while sufficiently retaining the desirable high conductivity and mechanical properties of graphene.^10^ Parylenes are widely used in the electronics industry as moisture barriers and dielectrics and have a plethora of advantages compared to other polymers, such as a highly controllable and uniform thickness, a physical vapor deposition process at ambient temperatures, and stability at elevated temperatures.^11^ Furthermore, a parylene-C coating has been shown to increase the conductivity of graphene due to doping from the chlorine atoms in the parylene-C.^12^

Recently, MEMS capacitive pressure sensors have received attention for having low power consumption, high sensitivity, and low temperature dependence when compared to piezoresistive pressure sensors. Ultra-thin (140 nm) GPH NEMS capacitive pressure sensors^13^ have been shown to rival both state-of-the-art metal–polymer and metal–silicon MEMS pressure sensors by combining high sensitivity and a wide operational pressure range. The general sensor design that is the focus of the current and previous study by Berger et al.^13^ consists of monolayer CVD graphene with a thin polymer support on top (GPH membrane) suspended over a large array of circular cavities in a SiO_2_ dielectric (Figure 2a). The suspended graphene acts as one deflecting capacitor electrode, and a doped Si substrate acts as the corresponding counter electrode. The GPH membrane is strongly clamped to the SiO_2_ substrate by van der Waals forces due to the large contact area between the membrane and surface. The low bending rigidity of the GPH membrane and the low surface roughness of SiO_2_ allow the membrane to conform closely to the substrate.^14^

One of the dichotomies in MEMS pressure sensor design is the trade-off between pressure sensitivity and dynamic pressure range—high sensitivity maximizes precision but typically at the expense of the operating pressure range. An accurate model of the deflection of NEMS membranes under pressure is essential to design membrane and cavity geometries that will deliver the targeted sensitivity and dynamic range for a given pressure-sensor application or similarly to design other NEMS devices to achieve pre-defined performance benchmarks. This can not be achieved in a time- and cost-effective manner by experimental trial and error. Previous studies modeling the deflection of graphene-based materials have mostly employed either pure-stretching or pure-bending approximations for simplicity. Both pure-stretching and pure-bending approximations overestimate the deflection since they only account for one source of resistance to deflection. Berger et al.^13^ employed a linear bending model to describe the deformation of their GPH membranes of thickness 160 nm since, when the deflection height *w*_0_ is small compared to the membrane thickness *t*, the system moves closer to the approximation that the deflection is bending rather than stretching dominated.^10,15^ Similarly, Cao et al.^16^ found that their 31 and 60 μm thick GPH membranes exhibited the pure-bending characteristic of linear maximum deflection with pressure. In a further study, Cao et al.^17^ showed that 22 μm thick GPH membranes acted with purely bending behavior prior to membrane delamination through measurement of the membrane deflection profile. Alternatively, for large deflections of thin membranes, the most commonly used pure-stretching description is Hencky’s power series solution^18^ which has been shown to be accurate for modeling monolayer graphene membranes^14,19,20^ in which the bending stiffness is negligible. Berger et al.,^10^ however, found that when increasing the thicknesses of GPH membranes from 20 to 207 nm while operating in the same pressure range, the mechanical regime shifts from pure stretching to pure bending behavior, indicating a need for an intermediate model that accounts for both effects and works in any deflection regime.

The Föppl–von Kármán (FvK) equations describe the static equilibrium mechanics of thin elastic plates, include both bending and stretching terms, and thus provide an accurate prediction of the deformation profile over the entire range of deflection.^21^ The equations are coupled, non-linear, fourth-order partial differential equations making them very difficult to solve analytically. Here, we employ a finite element method (FEM) to solve the FvK equations using *Oomph-lib*; an open source, object-oriented, multi-physics finite-element library.^22^ We compare the results to experimental 2D topographical deflection profiles as well as capacitance measurements to validate the accuracy of this method. Thus, we show that this type of computational simulation of thin-film membranes enables MEMS device designers to tailor their geometries to optimize device sensitivity, footprint, operating regime, and power consumption for a wide range of membrane thicknesses.

## Mathematical Methods

### FvK Equations

**Figure 1:**
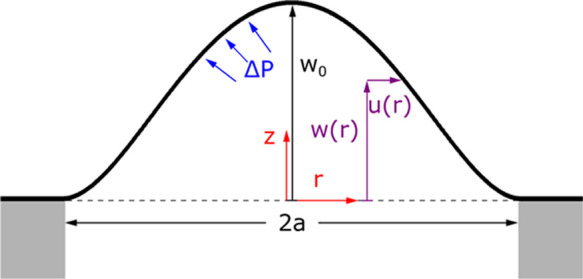
Cross-sectional schematic of an inflated axisymmetric membrane and pressure differential across the membrane.

The FvK equations describe the static equilibrium mechanics of thin isotropic elastic plates where the thickness of the plate, *t*, is much smaller than the characteristic in-plane length-scale of the plate, in this case, the radius *a* (i.e. *t*/*a* ≪ 1). As with other plate theories, they simplify the fully 3D elastic theory to one which describes a 2D surface embedded in 3D space by only modeling the midplane of the membrane. Mechanical equilibrium is described in terms of in-plane stresses (which are depth-averaged stresses) and bending stresses (which arise from variations in stress across the depth). This reduction to 2D theory is based upon the Kirchhoff–Love hypothesis (that normals to the midplane remain normal and do not change length) along with further kinematic assumptions of moderate rotation , and small strain .^23^ As the actuating part of the membrane is circular, we assume that our deformation is axisymmetric. In cylindrical polar coordinates (*r*, θ, *z*), the axisymmetric form of these equations is^24^
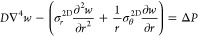
1
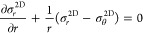
2where Δ*P* is the pressure differential across the membrane, ν is the Poisson ratio, σ_*r*_^2D^ and σ_θ_^2D^ are the in-plane stresses in the *r* and θ directions, respectively, *w* is the membrane deflection in the *z* direction, and *D* is the bending stiffness, Figure 1. The in-plane stresses σ_*r*_^2D^ and σ_θ_^2D^ are given in terms of the in-plane strains ε_*r*_, ε_θ_ by
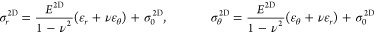
3where σ_0_^2D^ is the prestress, and *E*^2D^ is the 2D elastic modulus defined as the ratio of force-per-cross sectional length to strain in a sheet under uniaxial stress. The in-plane strains are given by
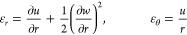
4where *u* is the radial displacement. Equation 1 is the force balance in the out of plane direction; the first term on the left arises from bending, the term on the left in brackets arises from in-plane stresses. Equation 2 is the radial force balance (the azimuthal force balance is trivially satisfied by the assumption of axisymmetry). Assuming the outer edge (*r* = *a*) is clamped, the displacements are subject to the following boundary conditions
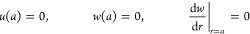
5

The symmetry at the center of the membrane implies
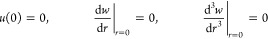
6

The weak form of these equations was solved using the FEM in *Oomph-lib*.^22^

### Limiting Regimes—Pure-Bending to Pure-Stretching

It is helpful to consider the nature of the FvK equations in regimes where pure-bending or pure-stretching models are considered appropriate in order to understand what makes them an improvement. If we denote the maximum deflection of the membrane by *w*_0_, the bending term in eq 1 scales as *P*_bend_ = *Dw*_0_/*a*^4^, growing linearly with deflection, whereas the stretching term scales as . If we compare the sizes of the two by taking their ratio
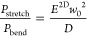
7we see that for a small deflection  the nonlinear stretching term contributes very little, and if we neglect it, we arrive at the equation for linear plate bending
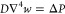
8which, with clamped boundary conditions, has the analytical solution
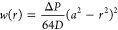
9

This solution is sufficient for very small deflection, but as pressure—and hence deflection—increases, the magnitude of the induced in-plane stresses grows until they become comparable to the bending stresses. In this moderate deflection regime, the linear bending model becomes inappropriate, and we require the full FvK equations to get an accurate picture. For very large deflections, however, (*w*_0_^2^ ≫ *D*/*E*^2D^), we can neglect the bending term to get the following equation
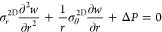
10which, when combined with eqs 2–4, describes a purely stretching sheet. In the case of a circular membrane pinned at the boundary (neglecting bending also requires us to abandon the fixed slope condition of clamping), there is an exact solution first published by Hencky,^25^ which assumes power series solutions for *w*, σ_*r*_^2D^, and σ_θ_^2D^. This membrane model closely resembles the FvK-based solution in the large deflection regime; however, large relative errors can be found near the boundary, where the behavior remains bending dominated.

### Membrane Parameters

For an effective predictive model, the behavior of the membrane should be predictable prior to construction. As such, to model a given membrane, we also require a system to obtain the parameters which appear in the FvK equations, one of which is a control parameter (σ_0_^2D^) and the remaining three are properties of the compound membrane (*E*^2D^, *D*, and ν). The system should only require knowledge of the membrane’s constitutive materials and composition—in the case of multi-layered membranes, it is sufficient to know each individual layer’s thickness *t*_i_, Poisson ratio ν_i_, and either the 2D modulus *E*_i_^2D^ or Young’s modulus *E*_i_.

The 2D elastic modulus of the membrane, *E*^2D^, can be found by summing the moduli of its layers *E*_i_^2D^. For a homogeneous isotropic material (such as parylene-C), *E*_i_^2D^ is equal to the 3D elastic modulus *E*_i_ multiplied by the layer thickness *t*_i_. Hence, the elastic modulus of our GPH membranes is
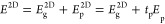
11

Multilayer membranes do not have a Poisson ratio in the same sense that homogeneous materials do; this is due to effects like inter-layer shear, delamination, and stress-induced bending moments brought on by the Poisson effect. By assuming the layers are perfectly bonded and that the moment induced by asymmetry is small, we can neglect the coupling between plane-strain and bending and arrive at a “mixing rule” for the effective Poisson ratio for the membrane (full derivation given in Note S1)
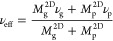
12where  and  are the 2D plane strain moduli of the graphene and parylene layers, respectively.^26^ The last parameter which belongs inherently to the membrane is the bending stiffness. For pure bending, there is always a neutral layer in which there is no in-plane stress: on one side of the neutral layer, the material is in compression, and on the other, it is in extension—this is the origin of bending stresses. In the case of homogeneous membranes, the neutral layer is in the middle, and the bending stiffness is given by^27^
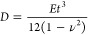
13

GPH membranes are multi-layered and hence non-homogeneous, so the bending stiffness is complicated by the neutral layer not necessarily being in the midplane and the stiffness varying throughout the depth. We know that the position of this neutral layer *z* = z* must be located such that the first moment of the plane strain modulus,  about z* is zero^28^
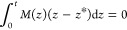
14

This implies
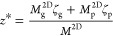
15where ζ_*i*_ is the location of the midplane of the *i* th layer. Given this, the bending stiffness, which is the second moment of the plane strain modulus about the neutral plane, is
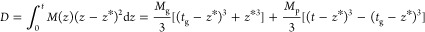
16

A full derivation of eq 15 is given in Note S2. Bending stiffness has in the past been approximated using (eq 13) using *E* = *E*^2D^/*t* and ν chosen by a volume mixing rule;^10^ however, asymmetry introduces error in the placement of z* and the concentration of stiffness near to/far from z* can dramatically decrease/increase *D*. Using eq 13 instead of eq 16 for GPH membranes underestimates stiffness by up to 25% for thick parylene-C layers and can overestimate *D* by as much as 800% for a 10 nm thick parylene-C layer.

### Capacitance Calculation

**Figure 2:**
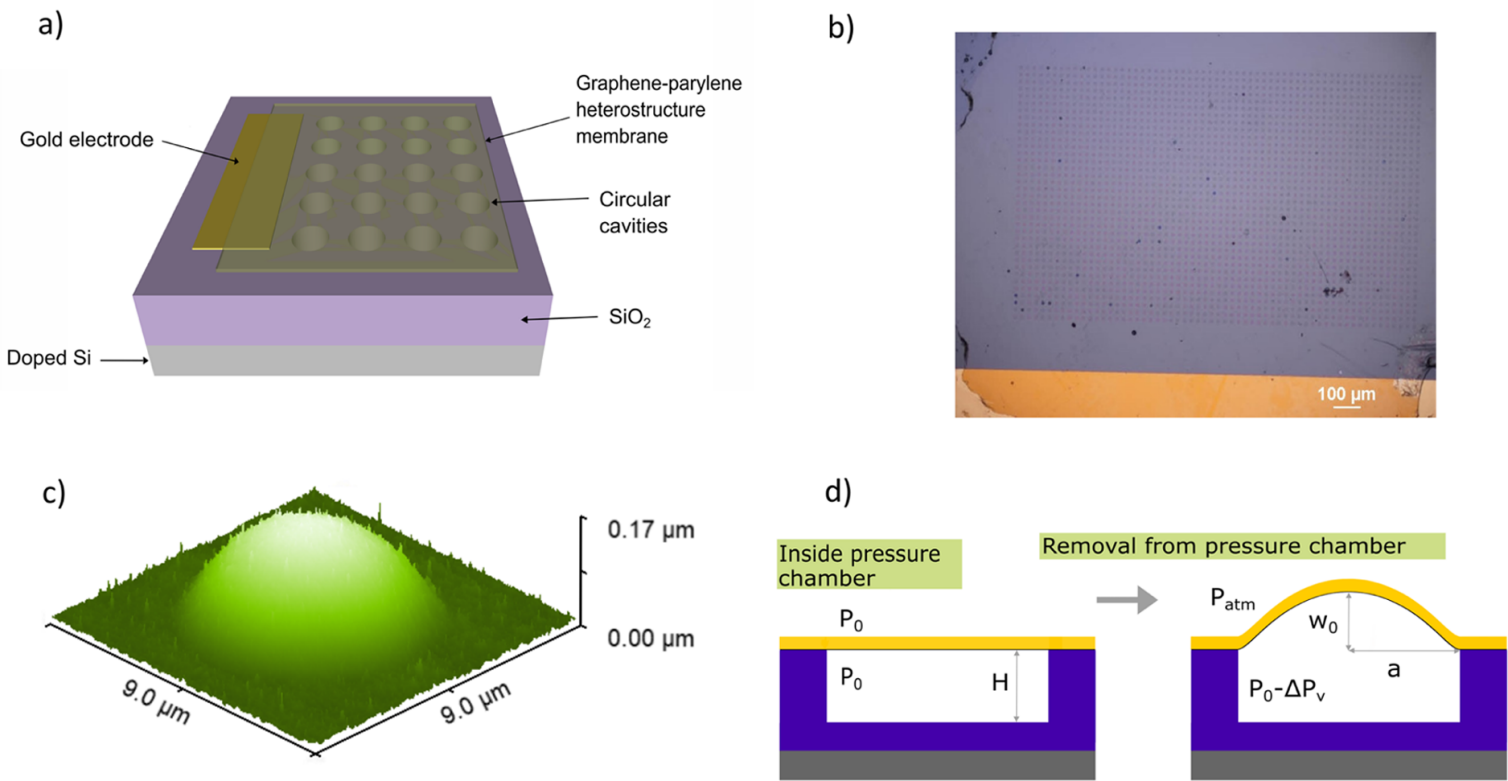
Design of sensor and micro-blister inflation testing. (a) 3D schematic of a GPH membrane capacitive pressure sensor. (b) an optical micrograph of the GPH membrane capacitive pressure sensor used to measure capacitance–pressure response, including gold electrodes, GPH membranes, and cavities. (c) 3D topographic atomic force microscopy (AFM) image of an inflated blister with radius *a* = 3.5 μm, thickness *t* = 45 nm, and subject to pressure Δ*P* = 0.94 kPa. (d) 2D schematic cross-section depicting a micro-blister inflation testing procedure with a single cavity and an actuating membrane, with significant parameters labeled.

In order to predict a device’s performance as a capacitive pressure sensor, it is necessary to know the capacitance at a specified pressure. As the capacitance of two surfaces depends only on their geometry and electric permittivity of the dielectric, we use the membrane deflection predicted by FvK as a function of the radial position, *w*(*r*) to find the capacitance of the actuating part, *C*_act_. To calculate the capacitance for a given deflection, we solve Gauss’ law for the electric potential under the assumption that the membrane deflection is slowly varying and use the resulting potential to find the charge density on the membrane (full derivation in Note S3), giving the capacitance across the air gap
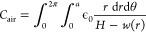
17where *H* is the cavity depth, depicted in Figure 2d. This is then combined with the capacitance across the SiO_2_ dielectric, given by *C*_ox_ = ϵ_ox_π*a*^2^/*t*_ox_, where *t*_ox_ and ϵ_ox_ are the thickness and permittivity of the SiO_2_ layer, respectively. Geometrically, as the electrodes are roughly parallel to one another, these parts can be combined as capacitors in series to yield the total capacitance of each actuating membrane
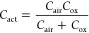
18

## Experimental Methods

### Fabrication of GPH Membrane Pressure Sensors

The GPH membrane pressure sensors, shown schematically in Figure 2a and by optical micrograph in Figure 2b, were fabricated via two process streams as depicted in Figure S1. Following Berger et al.,^13^ the first process was the fabrication of the monolayer graphene and the parylene GPH membrane with a poly(methyl methacrylate) (PMMA) and tape support enabling a dry transfer technique.^29^ The second was the fabrication of the sensor base, which incorporates an array of circular cavities etched into SiO_2_ on a doped Si substrate through photolithography and reactive ion etching. A rectangular gold electrode was deposited onto the SiO_2_ sensor base before the GPH membrane was transferred on top to allow electrical contact with the graphene on the underside of the GPH membrane. Finally, the GPH membrane was transferred onto the sensor base, and the PMMA and tape support were removed. The gold electrode was sandwiched between the SiO_2_ substrate and the graphene. An in-depth fabrication protocol is given in Note S4. The circular cavities used for micro-blister inflation had dimension radii *a* = 3.5, 5, and 7.5 μm and a depth of *H* ∼ 1000 nm. The cavities used for capacitance testing had *a* = 4.81 μm and *H* = 1077 nm (refer to Figure 2d). During device fabrication, a residual tensile prestress is introduced to the suspended GPH membranes. The prestress is difficult to control as it is a result of the mismatch of thermal expansion coefficients of the device materials.^30^

### Micro-Blister Inflation

Micro-blister inflation was conducted to provide an AFM profile to verify the FvK model and measure prestress in the membranes. Membranes of thicknesses of 45 ± 4, 65 ± 3, and 254 ± 10 nm were inflated to different degrees and imaged to provide several data points for comparison. The sensor was first placed inside a pressurized nitrogen chamber of pressure *P*_0_ > *P*_atm_. Within 24 h, the nitrogen diffuses through the SiO_2_, equalizing the external and internal pressures across the membrane; see Figure 2d. Upon removal of the device from the chamber into external pressure *P*_atm_, the GPH membranes inflate due to the pressure differential across them. The pressure differential across the membrane, Δ*P*, was calculated using Δ*P* = *P*_0_ – Δ*P*_v_ – *P*_atm_ where Δ*P*_v_ is the internal pressure change of an ideal gas due to the increase of internal volume. AFM in tapping mode was employed to map the 3D topographical surface of inflated micro-blisters (see Figure 2c), from which the radius and the maximum deflection height were measured, and 2D cross-sectional line profiles that intersect the maximum deflection point on the inflated membrane were extracted.

Prestress which arises during the fabrication process was measured using pressure-deflection data and analyzed with the spherical cap model which assumes that the pressurized sheet can be approximately modeled as a spherical cap under isotropic membrane strain, ε, and stress σ^2D^^10,31,32^
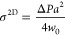
19
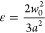
20where *a*, *w*_0,_ and Δ*P* are the cavity radius, membrane center deflection, and pressure differential across the membrane, respectively. Plots of stress against strain over a range of pressures were produced for membrane thicknesses of 45 and 65 nm. For isotropic biaxial stress, eq 3 yields
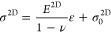
21and so the line of best fit for these plots has *y*-intercept at σ_0_^2D^. The slope of these lines also allows us to validate the predicted elastic modulus *E*^2D^. This model requires that *w*_0_ be in the range where *t* ≪ *w*_0_ ≪ *a* in order to both minimize bending effects and satisfy our kinematic assumptions. Our experiments were within this limit; therefore, we expect the error to be small. Further details of micro-blister inflation and measurement are given in Note S5 and Figure S2.

### Capacitance Testing

The variation of the capacitance with external pressure of a GPH device was measured and compared to that predicted by the numerical simulation. The device was placed in a custom-built pressure chamber pressurized with dry N_2_ gas, and the device capacitance was measured using an LCR meter (see Figure S3 for a set-up schematic and Note S6 for equipment details). The GPH device tested in this study is presented in the optical micrograph in Figure 2b. The GPH membrane used had *t* = 54 ± 4 nm. When capacitance measurements were taken, there were 1434 actuating membranes in total, obtained by the optical processing of Figure 2b. The focus of this comparative study was the change in device capacitance due to the actuation of the membranes—the change in capacitance of each actuating membrane Δ*C*_act_ was found by subtracting the baseline capacitance *C*_act_(Δ*P* = 0) from *C*_act_(Δ*P*). The total device capacitance change Δ*C* was found by multiplying Δ*C*_act_ by the number of actuating membranes, as the actuating units are in parallel with one another.

The pressure was cycled nine times between *P*_atm_ and a range of over pressures up to Δ*P* = 270 kPa; the mean Δ*C* and standard deviations were found for each Δ*P*. Each pressure cycle was 20 s, except for Δ*P* of 30, 60, and 80 kPa for which each cycle was 60 s to allow sufficient time for the chamber to reach the desired pressure, see Figure S4 for further information. These short timescales were adequate to negate the impact from N_2_ leakage.

## Results

### Micro-Blister Inflation Testing

Micro-blister inflation testing was performed on devices with membrane thicknesses of 45 and 65 nm. For both membranes, the maximum strain was no more than 0.5%. This is well within the linear elastic limits of graphene and parylene, which have been previously found to be 5.8^33^ and 1.2%,^34^ respectively. Figure 2c is a topographical surface map of an inflated micro-blister obtained by AFM, from which the blister height, radius, and 2D profile were extracted. The micro-blisters were found to deflate over time (Figure S2) due to the loss of N_2_ gas with a variability of deflation rates observed across individual membranes. Details of how this effect was accounted for are included in Note S5.

**Figure 3:**
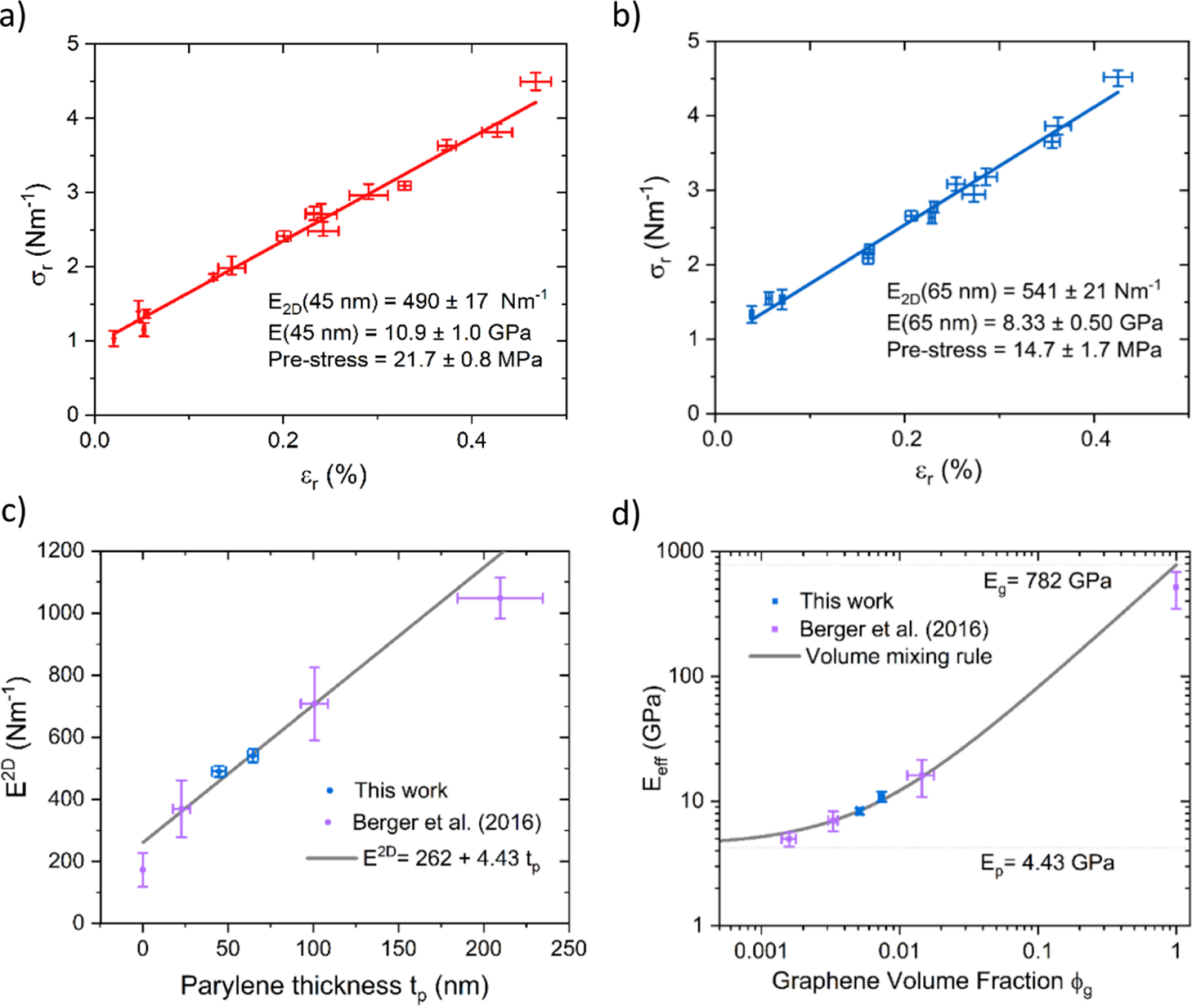
Micro-blister inflation testing results. (a,b) Radial stress–strain plots for GPH membranes of thicknesses of (a) 45 and (b) 65 nm with the quantities extracted from plots (*E*, *E*^2D^ and prestress) attained by fitting. The error bars are the combined measurement errors of *a*, Δ*P,* and *w*_0_ according to eqs 19 and 20. (c) Measured *E*^2D^ against parylene thickness with linear regression fit to eq 11. For data in this work, the *y*-error bars are from the uncertainties in linear regression from (a,b). The *x*-error bars are the standard deviation of seven thickness measurements. (d) Effective bulk modulus of GPH membranes as a function of the graphene volume fraction, fitted to the volume mixing rule (eq 22). The two horizontal dashed lines indicate the extracted bulk moduli of graphene and of parylene. The error bars were quantified by combining the error in the linear regression from (a,b) and the uncertainty in membrane thickness. The error bars for the data from Berger et al. (2016) were quantified by combining the stated uncertainties in the stated *E*^2D^ and membrane thickness uncertainties.

Using AFM data with spherical cap approximation, Figure 3a,b plots stress against strain using our pressure-deflection data for GPH membranes of thicknesses 45 nm (Figure 3a) and 65 nm (Figure 3b), respectively. Using ν = ν_eff_ from eq 12, this corresponds to a 2D modulus of *E*^2D^ = 490 ± 17 N m^–1^ for the 45 nm membrane and *E*^2D^ = 534 ± 21 N m^–1^ for the 65 nm membrane. The *E*^2D^ values are plotted against parylene thickness (Figure 3c), where we also include data from a study by Berger et al. (2016) that used the same materials and methodology. The straight line in Figure 3c was obtained by a fit to eq 11, with *E*_g_^2D^ = 262 ± 44 N m^–1^ and *E*_p_ = 4.43 + 0.74 GPa, which shows good agreement with the data collected.

By dividing these values by the membrane thickness, we arrive at the effective Young’s modulus which was found to be *E*_(45nm)_ = 10.9 ± 1.0 GPa and *E*_(65nm)_ = 8.33 ± 0.50 GPa. If we divide eq 11 through by *t,* we arrive at the mixing rule as used by Berger et al.^10^
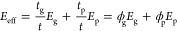
22which is shown in Figure 3d. The prestresses were found to be 0.95 ± 0.06 and 0.96 ± 0.07 N m^–1^ for the 45 and 65 nm membranes, respectively. The uncertainties in moduli and prestresses were obtained through linear regression analysis, with data weighted according to their errors. Using these constitutive material parameters along with the Poisson ratio of graphene ν_g_ = 0.21^35^ and the Poisson ratio of parylene ν_p_ = 0.40,^34^ we can describe any membrane constructed using graphene and parylene-C with eqs 11, 12, and 16.

### Comparison of AFM Profiles to Theory

**Figure 4:**
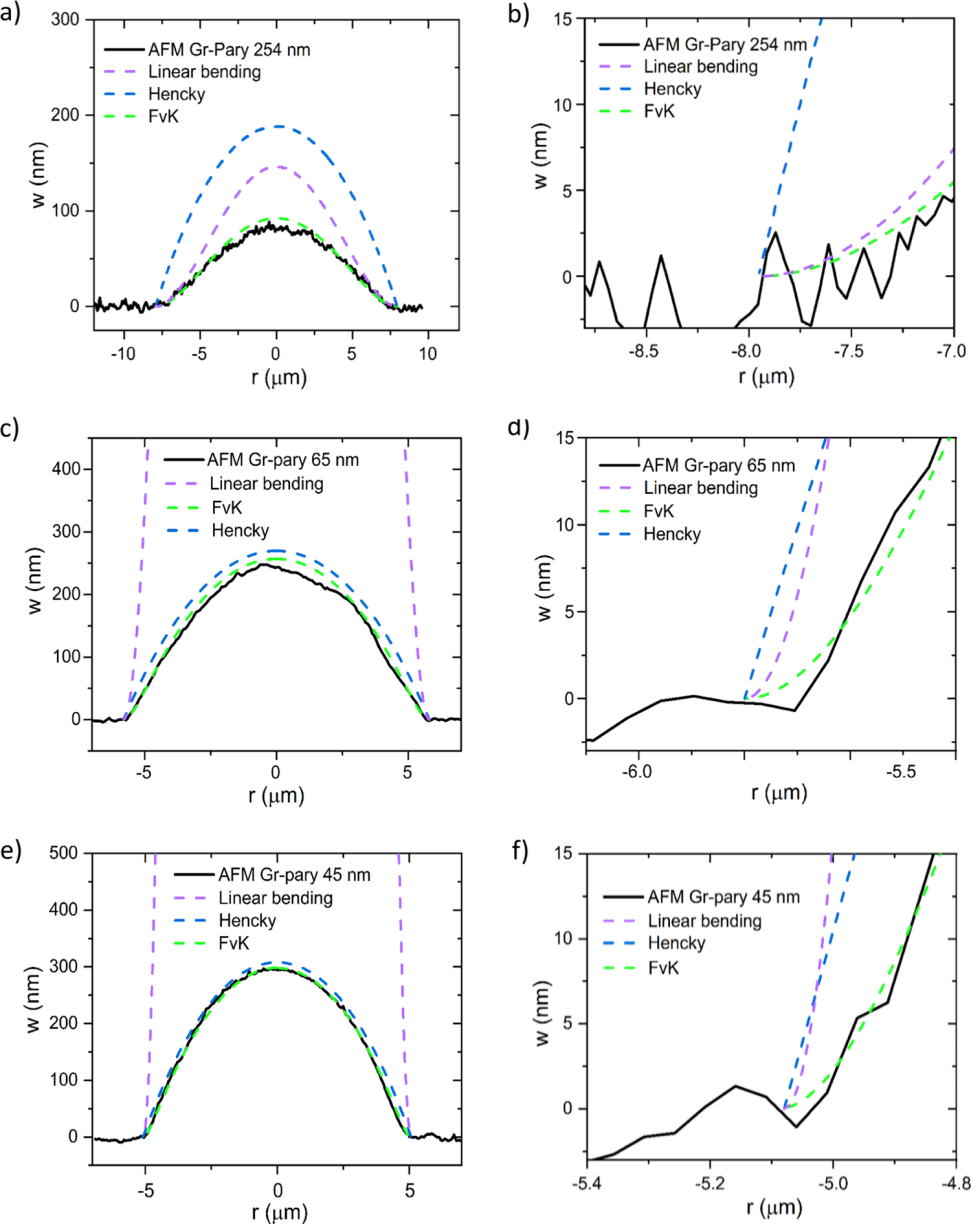
GPH membrane deflection against the radial position compared to linear bending, Hencky’s solution, and FvK models. (a) Membrane thickness of 254 nm pressurized to Δ*P* = 21 kPa. (b) Magnification of (a) around the clamped membrane edge. (c) Thickness of 65 nm pressurized to Δ*P* = 68 kPa. (d) Magnification of (b) around the clamped membrane edge. (e) Thickness of 45 nm pressurized to Δ*P* = 116 kPa. (f) Magnification of (e) around the clamped membrane edge.

Using the parameters presented in Table 1, we compared *Oomph-lib*-generated solutions of the FvK equations for a thin clamped circular membrane to the AFM 2D micro-blister cross-sections that intersect the maximum deflection point. As displayed in Figure 4, we compare the AFM profiles to FvK solutions, Hencky’s solution, and the linear bending model for membrane thicknesses of 45, 65, and 254 nm. In all cases, the 2D modulus of a GPH membrane was calculated using eq 11 using parameters obtained from the fitting, and the 2D prestress was always taken to be 0.96 N m^–1^. We find that neither Hencky’s solution nor a linear-bending equation can replicate the accuracy with which the FvK equations describe the GPH membranes.

**Table 1:** Predicted Membrane Parameters: a Table of Membrane Parameters for the Different Thicknesses of GPH Membranes Used in This Work, Calculated Using Eqs 11, 12, 15, and 16 and the Results in Figure 3c

thickness (nm)	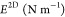	*E*_eff_ (GPa)	ν_eff_	D (pN m)
45	461	10.25	0.30	0.10
54	501	9.28	0.31	0.17
65	550	8.46	0.32	0.28
254	1390	5.46	0.37	11

In Figure 4a, we observe that both the linear bending and stretching models significantly overestimate the deflection height, demonstrating the need for a model that accounts for both sources of deflection resistance. In this deflection regime, where *w*_0_ ∼ *t*, bending and stretching forces are comparable in magnitude; it is here that only FvK performs adequately. By increasing the ratio *w*_0_/*t* (from Figure 4a to c to e), the deflection tends toward stretching dominated behavior: Hencky’s solution becomes more accurate, whereas linear bending significantly overestimates the deflection.

Figure 4b,d,f shows zoomed-in views near the boundary where pure-stretching models have large relative errors due to their inability to capture bending boundary layers. While Figure 4c,e demonstrates the small global error of Hencky’s solution to describe membrane deflection for large enough *w*_0_/*t*, Figure 4d,f presents the large local error at the clamped membrane edge, where bending effects remain important.

Importantly, FvK always outperforms both linear bending and Hencky’s solution globally and close to the boundary in all regimes as expected due to its inclusion of both bending and stretching forces. While our results capture the deflection regime in which the GPH membranes display mixed resistance as well as stretching-dominated behavior, our experiments were unable to resolve the regime in which they show close to pure-bending behavior. This would be in the regime where the ratio *w*_0_/*t* is much less than 1. This regime is difficult to assess experimentally; an increase in membrane thickness decreases the adhesion to the substrate and increases the gas leakage rate from the micro-blisters. Attempting to reduce the deflection by reducing the pressure is limited by the resolution of the pressure gauge and difficulty in measuring the deflection when the amplitude of deflection is comparable to that of the surface roughness. It has been seen before that increasing the thickness of parylene deposited increases the roughness of the surface.^36^

### Capacitance Comparison

Finally, we show that measured membrane capacitance can be successfully predicted using our simulation. The GPH capacitive device used for taking measurements is shown in the optical micrograph (Figure 2c) and has a membrane thickness of 54 nm.

**Figure 5:**
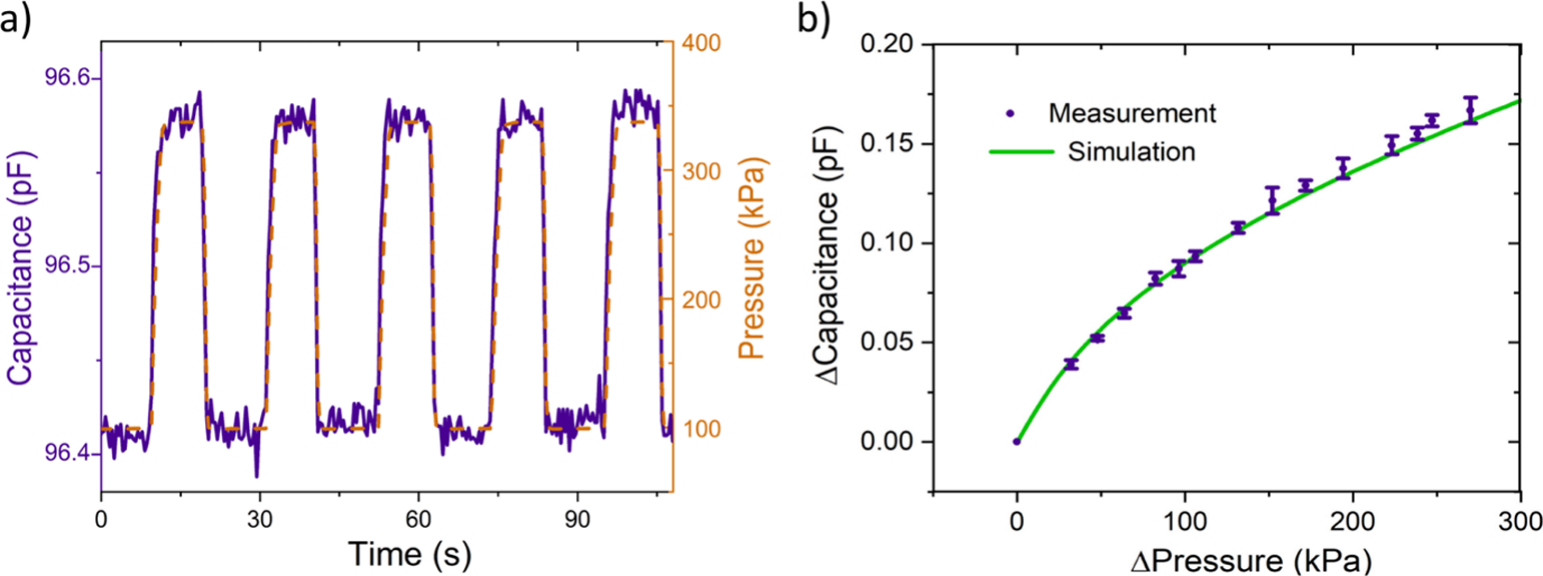
Capacitance–pressure response of the GPH capacitive pressure sensor. (a) Device capacitance and external pressure plotted against time over five pressure cycles from 340 kPa to atmospheric pressure. (b) Plot of average capacitance change as a function of pressure change, comparing FvK FEM simulation to measurement. The error bars are the standard deviation of nine readings.

Figure 5a shows the device capacitance and external pressure results from a representative pressure cycling run, where we alternated the external pressure between atmospheric pressure, 100, and 340 kPa. Figure 5a illustrates the promise of GPH membranes as fast response actuators with no measurable lag time when compared to the reference pressure gauge (MKS Baraton 772B pressure transducer). The main source of noise in the capacitance signal was the particulates in the silver paint used for electrical contact, as similar devices without silver paint have significantly reduced noise. The standard deviation of the noise was measured to be 0.006 pF, which corresponds to a theoretical resolution of this pressure transducer of 11 kPa. It has been shown that where the contact noise is lower, the sensitivity of GPH devices can be higher.^13^

Figure 5b illustrates the high accuracy of using the FvK simulation to predict Δ*C* for a specified value of Δ*P*. We see that the device sensitivity d*C*/d*P* is greatest at low pressures when bending is dominant and decreases as in-plane stresses increase.

## Conclusions

An accurate predictor of GPH membrane deflection mechanics is essential for achieving the required device sensitivity, operable pressure range, device footprint, and capacitance–pressure response. We showed that a numerical solution of the FvK equations allows accurate capacitance to pressure conversion for any cavity depth or radius. Furthermore, the limits of the operable pressure range can be determined by calculating the pressure at which the GPH membrane reaches the bottom of the cavity. The simulation also allows the determination of the stress and strain in the membrane, which can be used to predict the pressure at which elastic failure occurs. We have shown the effectiveness of an axisymmetric model, which can be extended to model membranes in non-circular shapes such as hexagons^13^ and squares.^9^ In conclusion, we have demonstrated the precision with which ultra-thin GPH NEMS membranes can be modeled by the FvK equations and solved using the open source, object-oriented, multi-physics finite-element library *Oomph-lib*. This method is more accurate than previously used models—the non-linear pure-stretching model and the linear pure-bending model. This model can be applied to GPH and other ultra-thin membrane NEMS such as pressure transducers, microphones, and ultrasound transducers.

## Supporting Information Available

Derivation of the effective Poisson’s ratio of a two-layer material; derivation of the location of the neutral plane of a two-layer membrane; derivation of capacitance across a slowly varying air gap; fabrication of graphene–parylene heterostructure pressure sensors; micro-blister inflation testing; capacitance–pressure response testing set-up; schematic of the two process streams used to fabricate graphene–parylene heterostructure pressure sensors; membrane deflation over time plot; and comparison of graphene–parylene heterostructure device capacitance–pressure response cycled to external pressure of 30 kPa overpressure with different time periods (PDF)

## Notes

All datasets and software used for supporting the conclusions of this article are available from the public data repository at the website.
