# Supplementary material for: Modeling Graphene–Polymer Heterostructure MEMS Membranes with the Föppl–von Kármán Equations
Source: ACS Appl Mater Interfaces. 2023 Feb 7;15(7):9853–61. doi: 10.1021/acsami.2c21096 (PMC9951177; doi:10.1021/acsami.2c21096)
Supplement: Supplementary file 1 — am2c21096_si_001.pdf [file am2c21096_si_001.pdf]

Supporting Information for:

Modelling graphene-polymer heterostructure MEMS membranes with the Föppl–von Kármán equations

Katherine Smith[1§], Aidan Retallick[2§], Daniel Melendrez[1], Aravind Vijayaraghavan[1,*], Matthias Heil[2]

[1]The University of Manchester, Department of Materials and National Graphene Institute, Manchester, M13 9PL, United Kingdom
[2] The University of Manchester, Department of Mathematics, Manchester, M13 9PL, United Kingdom
*****Corresponding author. Email: aravind@manchester.ac.uk

§K.S. and A.R. contributed equally to this paper

Note S1: Effective Poisson ratio of a two-layer material.

Throughout this section we use the superscript (i) to refer to the ith layer of the material when subscripts are already in use. For a two-layer membrane whose undeformed lower face is located at z_{l}, the upper face at z_{u} and the separating middle face at z_{m}, the 2D stress is:

$$\begin{aligned}\sigma_{ij}^{2\mathrm{D}}(x,y) &= \int_{z_{\mathrm{l}}}^{z_{\mathrm{u}}} \sigma_{ij}(x,y,z)\ dz \\ &= \int_{z_l}^{z_m} \sigma_{ij}(x,y,z)\ dz + \int_{z_m}^{z_u} \sigma_{ij}(x,y,z)\ dz \qquad \text{(SI 1.1)} \\ &= \sigma_{ij}^{2\mathrm{D}(1)}(x,y) + \sigma_{ij}^{2\mathrm{D}(2)}(x,y)\end{aligned}$$

where σ denotes the usual force-per-area found in bulk materials and $\sigma_{ij}^{2\mathrm{D}(k)}$ is the 2D stress in the kth layer.

The in-plane normal stresses in the y direction of each layer are given by (3) assuming no prestress:

$$\sigma_{yy}^{2\mathrm{D}(k)} = \frac{E_k^{2\mathrm{D}}}{1-\nu_k^2}\left(\varepsilon_{yy} + \nu_k \varepsilon_{xx}\right). \qquad \text{(SI 1.2)}$$

We wish to define an effective Poisson ratio which satisfies the traditional definition for a homogenous material. Hence if we apply uniaxial stress in the x direction we require that

$$\varepsilon_{yy} = -\nu_{\mathrm{eff}}\varepsilon_{xx}, \qquad \text{(SI 1.3)}$$

and from (SI 1.1) and the definition of uniaxial stress ($\sigma_{yy} = 0$) we have that

$$\sigma_{yy}^{2\mathrm{D}(1)} + \sigma_{yy}^{2\mathrm{D}(2)} = 0. \qquad \text{(SI 1.4)}$$

By substituting (SI 1.3) into (SI 1.2) we obtain that for each layer

$$\sigma_{yy}^{2\mathrm{D}(k)} = \frac{E_k^{2\mathrm{D}}}{1-\nu_k^2}(\nu_k - \nu_{\mathrm{eff}})\varepsilon_{xx}, \qquad \text{(SI 1.5)}$$

which in turn goes into (SI 1.4) to produce the equation

$$\frac{E_1^{2\mathrm{D}}}{1-\nu_1^2}(\nu_1 - \nu_{\mathrm{eff}})\varepsilon_{xx} = \frac{E_2^{2\mathrm{D}}}{1-\nu_2^2}(\nu_2 - \nu_{\mathrm{eff}})\varepsilon_{xx}. \qquad \text{(SI 1.6)}$$

By dividing through by ε_{xx} and rearranging for ν_{eff} we obtain

$$\nu_{\mathrm{eff}} = \frac{\dfrac{E_1^{2\mathrm{D}}}{1-\nu_1^2}\nu_1 + \dfrac{E_2^{2\mathrm{D}}}{1-\nu_2^2}\nu_2}{\dfrac{E_1^{2\mathrm{D}}}{1-\nu_1^2} + \dfrac{E_2^{2\mathrm{D}}}{1-\nu_2^2}} = \frac{M_1^{2\mathrm{D}}\nu_1 + M_2^{2\mathrm{D}}\nu_2}{M_1^{2\mathrm{D}} + M_2^{2\mathrm{D}}}, \qquad \text{(SI 1.7)}$$

where $M_k^{2\text{D}}$ is the 2D plane strain modulus of the kth layer.

Note S2: Neutral plane of a two-layer membrane

The first moment of the plane strain modulus about the neutral plane (the coupling moment) is required to be zero when the membrane is undergoing pure bending:

$$\int_0^t M(z)(z - z^*)\,\mathrm{d}z = 0\ . \tag{SI 2.1}$$

where $M(z)$ is the plane strain modulus at height z above the membrane bottom, t is the total thickness of the membrane and z^* is the height of the neutral layer.

This implies

$$\begin{aligned} z^* &= \frac{\int_0^t zM(z)\,\mathrm{d}z}{\int_0^t M(z)\,\mathrm{d}z} \\ &= \frac{\int_0^{t_1} zM_1\,\mathrm{d}z + \int_{t_1}^t zM_2\,\mathrm{d}z}{M^{2\text{D}}} \\ &= \frac{1}{M^{2\text{D}}}\left(\frac{M_1}{2}\left(t_1^2 - 0^2\right) + \frac{M_2}{2}\left(t^2 - t_1^2\right)\right) \\ &= \frac{1}{M^{2\text{D}}}\left(M_1 t_1 \frac{t_1}{2} + M_2(t - t_1)\frac{(t+t_1)}{2}\right) \\ &= \frac{M_1^{2\text{D}}\zeta_1 + M_2^{2\text{D}}\zeta_2}{M^{2D}}, \end{aligned} \tag{SI 2.2}$$

where t_1 is the thickness of layer 1 and ζ_i is the midplane of the ith layer.

Note S3: Capacitance across a slowly varying air gap

Here we orient our domain such that the bottom of the cavity is at $z = 0$, and the membranes, position is at $H - w(r)$.

To calculate the capacitance across dielectrics with permittivity ϵ, we must solve Gauss' law (assuming no unpaired charges)

$$\boldsymbol{\nabla} \cdot (-\epsilon \boldsymbol{\nabla} V) = 0, \tag{SI 3.1}$$

$$V|_{z=0} = 0, \qquad V|_{z=H-w(r)} = V_0, \tag{SI 3.2}$$

where V_0 is the potential difference across the capacitor electrodes. We then use the potential field V to calculate the surface charge density $q = \epsilon(\hat{\boldsymbol{n}} \cdot \boldsymbol{\nabla} V)$ on an electrode where $\hat{\boldsymbol{n}}$ is the unit normal vector to the electrode surface. Having done that, we integrate over the whole area to find the total charge

$$Q = \int q \, \mathrm{d}A = \int \epsilon(\hat{\boldsymbol{n}} \cdot \boldsymbol{\nabla} V) \, \mathrm{d}A, \tag{SI 3.3}$$

which, we can simply use in the definition of capacitance

$$C = \frac{Q}{V_0} = \frac{1}{V_0} \int \epsilon(\hat{\boldsymbol{n}} \cdot \boldsymbol{\nabla} V) \, \mathrm{d}A. \tag{SI 3.4}$$

As, across the air gap, $\epsilon = \epsilon_0$ is constant, we reduce (SI3.1) to Laplace's equation in an axisymmetric domain,

$$\nabla^2 V(r, z) = 0. \tag{SI 3.5}$$

If we assume that the surface of the deflected electrode is slowly varying such that $\frac{\partial w}{\partial r} \ll 1$ then we can also assume that $\frac{\partial V}{\partial r} \ll 1$ and, neglecting edge effects, Laplace's equation simplifies to

$$\frac{\partial^2 V}{\partial z^2} = 0, \tag{SI 3.6}$$

which results in a parallel field approximation

$$V(r, z) = A(r) z + B(r). \tag{SI 3.7}$$

The bottom boundary condition at $z = 0$ in (SI 3.2) requires $B(r) \equiv 0$ and the condition on the upper membrane boundary gives us $A(r)$ such that

$$V(r, z) = \frac{V_0}{H - w(r)} z. \tag{SI 3.8}$$

Using SI 3.4, integrating over the surface at the bottom of the cavity, where $\hat{\boldsymbol{n}} = \hat{\boldsymbol{k}}$ and $\boldsymbol{\nabla} V = V_0 / (H - w(r)) \hat{\boldsymbol{k}}$, the charge density on the flat electrode at the cavity bottom is

$$C_{\mathrm{air}} = \int \epsilon_0 \frac{\mathrm{d}A}{H - w(r)} = \int_0^{2\pi} \int_0^{a} \epsilon_0 \frac{r \, \mathrm{d}r \mathrm{d}\theta}{H - w(r)}. \tag{SI 3.9}$$

Note S4: Fabrication of graphene-parylene heterostructure pressure sensors

Chemical vapour deposition (CVD) graphene grown on 25 µm thick copper foil and was purchased from BGT Materials. The topside CVD graphene was spin coated with 200 nm thick PMMA for support and the underside graphene was exposed to a low power O_2-Ar plasma for removal. The copper was etched from underneath using 0.1 M ammonium persulfate solution. Once the copper was fully etched, the floating graphene-PMMA membrane was transferred to 3 consecutive deionised (DI) water baths, with a rinse time of 30 minutes each. The graphene-PMMA stacks were wet transferred onto blank SiO_2/Si, dried with nitrogen and heated up to 130 °C for 1 hour. The PMMA was removed by submerging in acetone for 2 hours, followed by a rinse in hexane.

A thin layer of Parylene-C (di(2-chloro-p-xylylene)) was then deposited onto the graphene on SiO_2 via chemical vapour deposition using a SCS Labcoater 2010 system with an Inficon XTC2 deposition controller. The pyrolysis furnace is heated to 690°C and the deposition chamber set to 15 mBar. After Parylene deposition, a 200 nm PMMA layer is spin coated onto the top side and a low-residue tape window is pressed onto the stack, providing structural support for the membrane stack. The SiO_2 and Si are etched away using 0.25 M potassium hydroxide solution. The membranes are again rinsed with 3 consecutive DI water baths and then attached onto a stainless steel plectrum, using double sided sticky tape. After 30 minutes of drying at room temperature the graphene-Parylene-PMMA membranes are ready for dry-transfer onto the cavity patterned substrate.

The cavity patterned substrate was produced by firstly depositing a thin 50 nm layer of Al onto a SiO_2/ Si wafer (SiO₂ thickness 1500 nm, Microchemicals), through thermal evaporation with Ohmic heating with a MiniLab Moorfield system. Next, Microposit™ S1813™ photoresist (PR) polymer of thickness 2 µm was spin coated onto the Al and baked for 2 minutes at 110 °C. The cavity pattern with circles of diameter 7, 10 and 15 µm was exposed using a 405 nm GaN laser and the exposed PR was removed with MF319 (Microchem) developer. The Al was removed from the exposed circles using Aluminium etchant, leaving a hard mask for etching deep (~1000 nm) cavities into SiO_2 via reactive ion etching (RIE). The S1813 and Al were removed and the graphene-Parylene-PMMA membrane was transferred onto the substrate.

Vacuum ports were used to attach the plectrum to a transfer arm and the cavity patterned substrate to a hot plate. The substrate is heated to 60°C and the plectrum lowered until the membrane is in contact with the substrate. The membrane was laminated onto the substrate and the tape removed using a sharp tool to tear the graphene-Parylene-PMMA membrane. The membrane is then heated to 130 °C for 10 minutes to increase adhesion to the substrate. Finally, the PMMA was removed by submerging in acetone for 2 hours, followed by a rinse in hexane.

The membrane was suspended over the cavities and also laminated onto a rectangular 2 nm/50 nm chromium/gold electrode which was deposited by thermal evaporation (Moorfield MiniLab), the purpose of which is to create electrical contact between the underlying graphene and a wire bonded to the gold. A wire was also bonded to the doped Si base to allow capacitance measurements between the graphene and silicon to be taken.

Note S5: Micro-blister inflation testing

The 3D topographical map was measured through Atomic Force Microscopy (AFM). The system was a Bruker Dimension FastScan with FastScan-A silicon nitride tips. The measurements were carried out in ScanAsyst™ Tapping Mode and a peak force set at 35 nN, to reduce damage to the membranes.

The micro-blisters were inflated by pressurizing them with N_2 gas in a chamber equipped with a bourdon spring pressure gauge (SMC, range 0 - 4 bar). The membranes were cycled between 0 kPa and 100 kPa to partially release pre-tension from the fabrication process. The samples were left for at least 24 hours to allow the N_2 to diffuse into the cavities through the SiO_2 layer, until the pressure across the membrane equalises. The blisters are then removed from the chamber (at time $t = 0$) and an AFM image is produced within 3 minutes of the return to external atmospheric pressure. AFM images are produced every 2 minutes for 25 minutes to record the deflation over time, from which the blister height before deflation is extrapolated.

To calculate the radial stress, σ_r, it is required to determine the pressure differential across the membrane ΔP. The internal pressure in the cavity P_0 is greater than P_{atm}, causing the membrane to inflate and the volume beneath the membrane increases. Since the rate of N_2 diffusion is slow, we assume the number of N_2 particles is constant which leads to application of Boyle's law to find the internal pressure reduction, ΔP_{V}, due to internal volume change

$$P_0 - \Delta P_V = \frac{P_0}{1 + \frac{w_0}{3H}} \qquad \text{(SI 5.1)}$$

where w_0 is the maximum deflection height.

To calculate an accurate value of maximum deflection height at t = 0, the deflation (see Supplementary Figure 2) that occurred between removal of the device from the pressurization chamber and the AFM measurement was accounted for. The time between removal and measurement was 3-5 minutes. This effect was accounted for by taking 3-d AFM images every 2-3 minutes for between 20-25 minutes, plotting $\ln w_0$ against time and conducting a linear regression fit. Figure 2a shows the good fit of the AFM height measurement data against time with an exponential decay curve. Figure 2b shows the good fit of $\ln w_0$ against time with a straight line. The w_0 at $t = 0$ was extrapolated from the equation of the line and the error in the y-intercept was used to find the error in w_0.

We calculated accurate values of P_0 to use in the FvK simulations to compare to AFM defelction profiles using by applying the membrane characteristic $w_0 \propto P^3$.

Note S6: Capacitance-pressure response testing set-up

The sample chamber was fitted with a commercial reference pressure sensor (MKS Baratron 772B) and an electronic valve (MKS instruments T3Bi). The gas inlet pressure was varied using an Aventics pneumatic pressure control valve. The LCR meter used to measure device capacitance was an Iso-tech LCR 819. The measurements were carried out at a 1 kHz test frequency and a 1 V voltage bias. The sample rate of pressure and capacitance data acquisition was 2.5 Hz. A custom graphic user interface (GUI) for data capture and equipment control was written in Matlab 2019b and used to communicate with the LCR meter and the electronic valve from our pressure system. The GUI controlled the open/close cycling of the electronic valve and captured both pressure gauge and LCR meter readings through XML-based scripts.

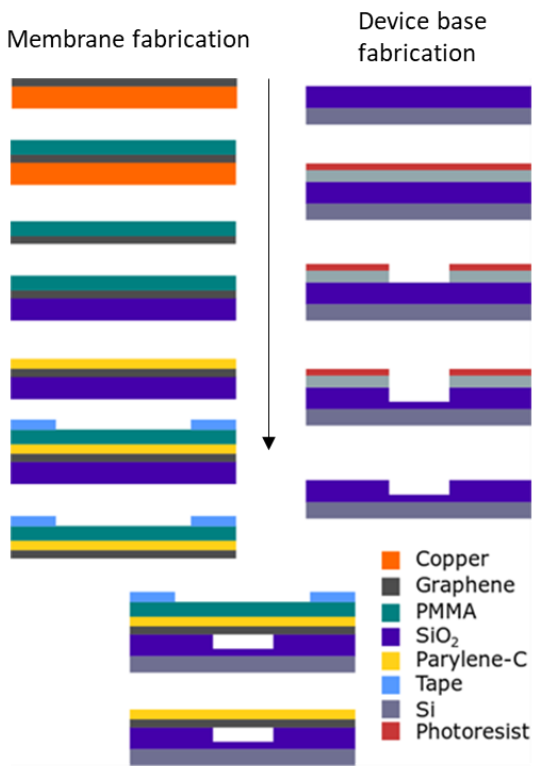

Figure S1 – **A schematic of the 2 process streams used to fabricate the graphene-parylene heterostructure pressure sensors.** Description of each step is given in Note S4.

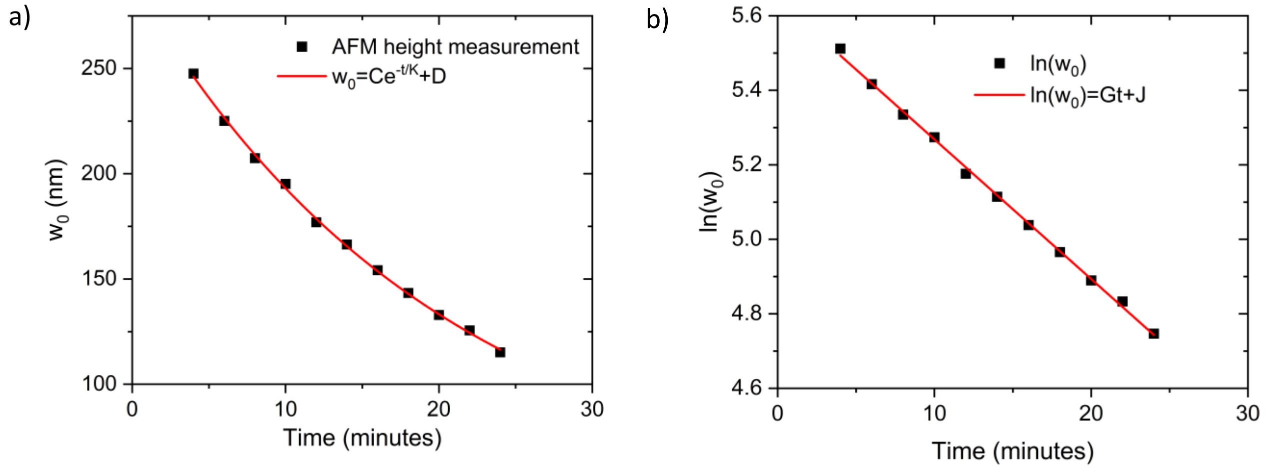

Figure S2- Deflation over time of a 10 μm diameter, 65 nm thick membrane inflated with an initial internal pressure P=181 kPa. (a) Maximum deflection height (w_0) againt time. The solid line is the best fitting exponential decay curve $w_0 = Ce^{-t/K} + D$ where C,D and K are constants. Fitted with the Levenberg Marquardt iteration algorithm. (b) $ln(w_0)$ against time. The solid line is a linear line of best fit of the equation $ln(w_0) = Gt + J$. Fitted with linear regression analysis.

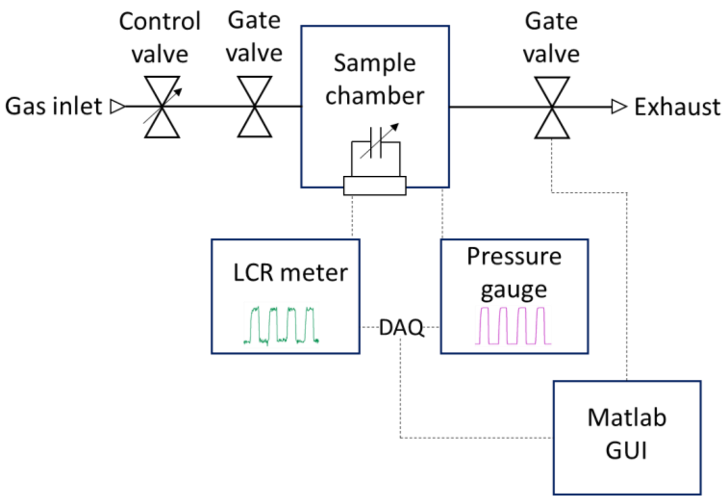

Figure S3 – Schematic of the measurement set-up used to measure the capacitance and external pressure of GPH capacitive pressures sensors described in Note S6.

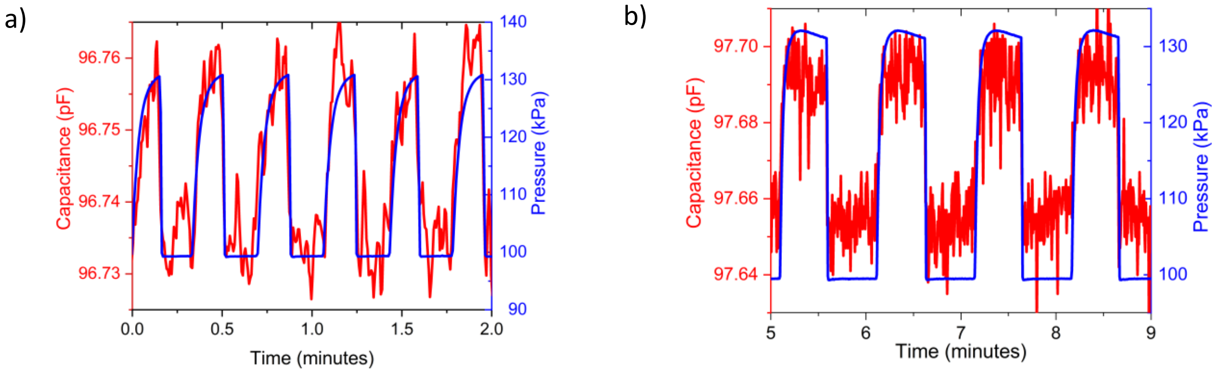

Figure S4- Comparison of graphene-parylene heterostructure device capacitance-pressure response cycled to external pressure of 30 kPa over pressure with different time periods. a) Cycle period of 20 seconds. b) Cycle period of 60 seconds. At first each, each pressure cycling run was conducted with a period of 20 seconds. It was found that 10 seconds pressurization time of the external chamber was appropriate for higher ΔP values, as the pressure differential between the chamber pressure and gas inlet was sufficient to cause a high gas flow rate and short pressuriation time. However, for low ΔP, the gas flow rate was too slow for the chamber pressure to equalize the inlet pressure (Figure S4a). To combat this, for $\Delta P =$ 30, 60 and 80 kPa, the cycle period was changed to 60 s, and as shown in Figure S4b, the allows sufficient time for the external chamber to reach the required pressure.
